# Benchmarking Transfer Entropy Methods for the Study of Linear and Nonlinear Cardio-Respiratory Interactions

**DOI:** 10.3390/e23080939

**Published:** 2021-07-23

**Authors:** Andrea Rozo, John Morales, Jonathan Moeyersons, Rohan Joshi, Enrico G. Caiani, Pascal Borzée, Bertien Buyse, Dries Testelmans, Sabine Van Huffel, Carolina Varon

**Affiliations:** 1 STADIUS, Center of Dynamical Systems, Signal Processing and Data Analytics, Department of Electrical Engineering (ESAT), KU Leuven, 3001 Leuven, Belgium; jmorales@esat.kuleuven.be (J.M.); jonathan.moeyersons@esat.kuleuven.be (J.M.); sabine.vanhuffel@esat.kuleuven.be (S.V.H.); carolina.varon@esat.kuleuven.be (C.V.); 2 Department of Patient Care and Monitoring, Philips Research, 5656 AE Eindhoven, The Netherlands; rohan.joshi@philips.com; 3 Department of Electronics, Information and Bioengineering, Politecnico di Milano, 20133 Milan, Italy; enrico.caiani@polimi.it; 4 Department of Pneumology, Leuven University Centre for Sleep and Wake Disorders, UZ Leuven, 3000 Leuven, Belgium; pascal.borzee@uzleuven.be (P.B.); bertien.buyse@uzleuven.be (B.B.); dries.testelmans@uzleuven.be (D.T.); 5 Service de Chimie-Physique E.P., Université libre de Bruxelles, B-1050 Brussels, Belgium

**Keywords:** transfer entropy, surrogate data, cardio-respiratory interactions, polysomnography

## Abstract

Transfer entropy (TE) has been used to identify and quantify interactions between physiological systems. Different methods exist to estimate TE, but there is no consensus about which one performs best in specific applications. In this study, five methods (linear, k-nearest neighbors, fixed-binning with ranking, kernel density estimation and adaptive partitioning) were compared. The comparison was made on three simulation models (linear, nonlinear and linear + nonlinear dynamics). From the simulations, it was found that the best method to quantify the different interactions was adaptive partitioning. This method was then applied on data from a polysomnography study, specifically on the ECG and the respiratory signals (nasal airflow and respiratory effort around the thorax). The hypothesis that the linear and nonlinear components of cardio-respiratory interactions during light and deep sleep change with the sleep stage, was tested. Significant differences, after performing surrogate analysis, indicate an increased TE during deep sleep. However, these differences were found to be dependent on the type of respiratory signal and sampling frequency. These results highlight the importance of selecting the appropriate signals, estimation method and surrogate analysis for the study of linear and nonlinear cardio-respiratory interactions.

## 1. Introduction

Physiological signals are observations of complex processes resulting from the combination of their internal dynamics, their interactions with other processes and third party effects, such as the influence of medication and treatments. These interactions might occur in a linear and/or a nonlinear fashion. The development of methods to evaluate and quantify these interactions is currently an active research topic.

Several methods to identify the interactions between complex processes have been developed across different fields, and many of them have been adapted to the study of physiological data [1,2,3,4,5,6,7,8,9,10]. One of these approaches, widely used for the detection of information flow between processes, is Transfer Entropy (TE). TE is able to identify linear and nonlinear interactions, depending on its implementation. It is also able to identify the directionality of the interactions [11]. TE relies on the past observations of the processes, and can be considered as a parametric or a non-parametric approach according to the assumptions made for its computation. For its numerical estimation, two factors need to be considered. First, an embedding technique to generate vectors describing the past of the processes and, second, an entropy estimator [11,12,13,14]. There are several entropy estimators differing in assumptions, advantages and limitations, with no consensus about which one performs best [15]. For this reason, the selection of the best method to estimate TE for each specific application is still an open problem.

A first aim of this study is to compare five of the most commonly used methods for TE computation, in order to provide a framework to support the selection of the best approach to be applied in short physiological signals to identify and quantify linear and nonlinear interactions. Each method uses a different entropy estimator (linear [12,16,17], k-nearest neighbors estimator [12,18], fixed-binning with ranking [13,15], kernel density estimator [13,19,20], and adaptive partitioning [13,15]). In all the implementations, a uniform embedding technique is chosen to generate the embedding vectors [11,14].

Each method is hypothesized to perform differently according to the tuning of their parameters and the characteristics of the interactions between the processes in which they are applied [21]. Considering this, three simulation models are proposed: linear [16], nonlinear [13], and linear + nonlinear [22]. Two analyses were conducted for each of these simulations. The goal of the first analysis was to find the best set of parameters for each method and to observe their potential to identify the correct lag at which the interactions occur. The second analysis studied the behavior of the methods when modifying the strength of the interactions between the processes. The performance was evaluated in the simulations using Transfer Entropy Excess (TEE), a new index proposed in this study.

A second goal of this study is to use the proposed framework to select the best method and apply it on clinical data. Specifically, on polysomnography (PSG) recordings, to test the hypothesis that the linear and nonlinear components of the cardio-respiratory interactions during light (NREM1) and deep (NREM3) sleep change with the sleep stage.

Previous studies have shown that the linear and nonlinear dynamics in both cardiac and respiratory signals change with the sleep–wake cycle and between sleep stages [6,7,15,16], and that these changes affect both signals similarly [23,24,25]. This fact raised some questions regarding how these changes affect the cardio-respiratory interactions during sleep, which led to the identification of three forms of coupling, namely, respiratory sinus arrhythmia (RSA), cardio-respiratory phase synchronization (CRPS), and time delay stability (TDS) [6]. In this study, TE was used to identify and quantify the cardio-respiratory interactions during sleep stages, using the best method for its computation found with the simulation models.

To the best of the knowledge of the authors, no study exists that compares all five methods presented here for the computation of TE. However, previous works have studied the performance of some of these methods, in particular [12] and [26] comparing LIN and KNN, [13] comparing FBR, KDE and DVP, and [27] comparing KNN and a KDE with a different kernel function.

In addition, the way this study quantifies and identifies linear and nonlinear interactions during light and deep sleep has not been found elsewhere in the literature. The results of this study could serve as a base for the selection of suitable TE methods for the analysis of linear and nonlinear interactions in physiological systems.

This paper is divided as follows: Section 2 introduces the used methods and datasets. Section 3 shows the results for the TE computation on the simulation models and the clinical data. Section 4 discusses the results. Finally, Section 5 concludes this work.

## 2. Materials and Methods

This section describes the theory behind Transfer Entropy (TE) and the factors to be considered for its computation. Following these descriptions, the simulation study and the application to clinical data are explained.

### 2.1. Transfer Entropy

Consider the stochastic process *Y* = $\left\{y_{1},y_{2},y_{3},\ldots ,y_{N}\right\}$, with *N* the total number of observations of the process. The Shannon Entropy can be defined as the average information content of the process [11,28]. This can be computed as

(1) $$H\left(Y\right)=-\sum_{y\in Y}p\left(y\right)log\left(p\left(y\right)\right),$$

where p(y) is the probability mass function (PMF) of the observation *y*. The base of the logarithm determines the units of *H*. When using the natural logarithm (log), *H* is measured in nats.

Now, consider a second stochastic process *X* = $\left\{x_{1},x_{2},x_{3},\ldots ,x_{N}\right\}$, which has a joint probability distribution with *Y*. The joint information provided by both processes is described by the Joint Entropy, as

(2) $$H\left(X,Y\right)=-\sum_{y\in Y}\sum_{x\in X}p\left(x,y\right)log\left(p\left(x,y\right)\right),$$

where p(x,y) is the joint PMF of the observations *x* and *y*.

The remaining uncertainty for the observations of *Y* given the information of *X* is described by the Conditional Entropy as

(3) $$H\left(Y|X\right)=-\sum_{y\in Y}\sum_{x\in X}p\left(x\right)p\left(y|x\right)log\left(p\left(y|x\right)\right),$$

where p(y|x) is the conditional PMF of the observation *y* given the observation *x*.

Transfer Entropy (TE) estimates the reduction of uncertainty of the observations of *Y* (target process), accounted by both the past observations of *X* (driver process) and the past observations of *Y*, compared to the reduction of uncertainty of the observations of *Y* accounted only by its past [29]. TE can be defined in terms of joint or equivalently in terms of conditional entropies as

(4) $$\begin{array}{cc} TE_{X\overset{}{\to }Y}= & H\left(Y,Y^{-}\right)-H\left(Y^{-}\right)-H\left(Y,X^{-},Y^{-}\right)+H\left(X^{-},Y^{-}\right), \end{array}$$

$$\begin{array}{cc} H(Y,X^{-},Y^{-})= & H\left(X^{-},Y^{-}\right)+H\left(Y|X^{-},Y^{-}\right),\\ H(Y,Y^{-})= & H\left(Y^{-}\right)+H\left(Y|Y^{-}\right), \end{array}$$

(5) $$\begin{array}{cc} TE_{X\overset{}{\to }Y}= & H\left(Y|Y^{-}\right)-H\left(Y|X^{-},Y^{-}\right), \end{array}$$

where $H(Y,Y^{-})$ is the joint entropy of *Y* and its past, $H(Y,X^{-},Y^{-})$ is the joint entropy of *Y* and the past of both processes, and $H(X^{-},Y^{-})$ is the joint entropy of the past of both processes. $H(Y|Y^{-})$ is the conditional entropy of *Y* given its own past, and $H(Y|X^{-},Y^{-})$ is the conditional entropy of *Y* given its own past and the past of *X*. Given this dependency on the past observations of both processes, TE is an asymmetric measure, which constitutes an advantage when quantifying the directionality of the information transfer from one system to the other [11,30].

Considering the definition of TE, two factors are relevant for its computation: the definition of the vectors describing the past observations, or so-called embedding vectors, of the processes; and the estimation of the entropies. Depending on the estimation of entropies, TE accounts for linear and/or nonlinear interactions [14].

### 2.2. Definition of Embedding Vectors

From the observations of the driver and target processes, embedding vectors can be defined to describe their past to estimate the TE [11,12,13,14,31].

The selection of the elements of the embedding vectors is important, given that they should contain the most relevant past observations of the processes. Sub-optimal selection might be problematic. Too long embedding vectors will lead to redundancy as well as increased computational requirements, and too short vectors will lead to insufficient information. In order to tackle this challenge, several embedding techniques can be used. The uniform embedding (UE) is the most common technique used in the computation of TE because of its simplicity. For this reason, in this study, this technique in combination with different entropy estimators is used.

For UE, the embedding vectors are constructed as $X_{t}^{m}=\left\{X_{t-\tau },X_{t-2\tau },\ldots ,X_{t-(m-1)\tau },X_{t-m\tau }\right\}$ and $Y_{t}^{n}=\left\{Y_{t-\tau },Y_{t-2\tau },\ldots ,Y_{t-(n-1)\tau },Y_{t-n\tau }\right\}$, where τ is the time lag, and *m* and *n* are the embedding dimensions.

### 2.3. Estimation of Entropies

The exact estimation of the PMF in an analytical way is an open research problem. However, there is a wide range of options to estimate the PMFs, depending on the application and the characteristics of the processes to analyze [11]. In this study, five entropy estimators that are implemented in the open source tools by Montalto et al. [12] and Lee et al. [13] were considered: linear (LIN); k-nearest neighbors (KNN); fixed-binning with ranking (FBR); kernel density estimation (KDE); and adaptive partitioning (DVP).

#### 2.3.1. Linear Estimator

The linear estimator (LIN) assumes that the processes *X* and *Y* have a joint Gaussian PMF. This assumption allows for working with exact expressions already derived for the entropy measures [12,16]. The entropy of a Gaussian random process *Y* can be expressed as

(6) $$H\left(Y\right)=\frac{1}{2}\log\left(2\pi e\Sigma (Y)\right),$$

where Σ(Y) is the covariance of *Y*, and *e* is the Euler’s constant. Now, the conditional entropy of *Y* given *X* is defined as

(7) $$H\left(Y|X\right)=\frac{1}{2}\log\left(2\pi e\Sigma (Y|X)\right),$$

where Σ(Y|X) is the partial covariance of *Y* given *X*, which corresponds to the variance of the residuals of a linear regression of *Y* on *X*. Considering this, the conditional entropy can be rewritten as

(8) $$H\left(Y|X\right)=\frac{1}{2}\log\left(|\Sigma (\epsilon )|\right)+\frac{1}{2}N\log\left(2\pi e\right),$$

where ϵ are the residuals of the linear regression [17]. Finally, the TE is computed by implementing (8) in (5). More details about this approach can be found in [17].

#### 2.3.2. K-Nearest Neighbors Estimator

The k-nearest neighbors (KNN) estimator approximates the PMF by calculating a local probability mass in the neighborhood of each of the observations of the process *Y*. The probability mass is estimated by considering a sphere whose diameter is equal to twice the expected distance to the k-th nearest neighbor of each observation.

Following the procedure presented in [18], H(X,Y) can be computed as

(9) $$H\left(X,Y\right)=-\psi \left(k\right)+\psi \left(N_{X,Y}\right)+\log\left(c_{d_{X}}c_{d_{Y}}\right)+\frac{d_{X}+d_{Y}}{N_{X,Y}}\sum_{i=1}^{N_{X,Y}}\log\left(p(\zeta (i))\right),$$

where ψ is the digamma function, $N_{X,Y}$ is the number of observations in the joint space [X,Y], $d_{X}$ and $d_{Y}$ are the dimensions of *X* and *Y*, $c_{d_{X}}$ and $c_{d_{Y}}$ are the volumes of the $d_{X}$-dimensional and $d_{Y}$-dimensional unit spheres, respectively, and ζ(i) is twice the distance from a point ($x_{i},y_{i}$) to its k-th nearest neighbor. TE is computed applying (9) to estimate the entropies in (4). More details about this method are given in [12,18].

#### 2.3.3. Fixed-Binning with Ranking Estimator

The fixed-binning estimation is one of the approaches most widely used for PMF approximations due to its simplicity. This method performs a uniform quantization of the observations of the processes in *Q* bins, after normalizing the values to zero mean and unit variance. With this, the approximation of the probabilities, *p*, is done based on the relative frequencies of occurrence of each quantized state,

(10) $$p\left(g_{i}\right)=\frac{r_{i}}{N},$$

where $g_{i}$ is the event associated with a specific bin *i* ($X,X^{-},Y^{-}$ and their combinations) and $r_{i}$ is the number of observations that lie in the bin *i*.

The fixed-binning method faces challenges related to robustness, convergence with high dimensionality, and the selection of the number of bins to be used when quantizing the time series. To improve the robustness of this method, some modifications have been proposed [13,15]. One of them consists of an ordinal sampling, also known as ranking. For the fixed-binning with ranking (FBR) estimator, the time series of the observations of *X* and *Y* are replaced by two new series *U* and *V*, respectively. The values in *U* and *V* are integers ranging from 1 to *N*, representing the rank of the corresponding observations of *X* and *Y*. After the ranking, the PMFs are estimated using (10) in the space defined by *U* and *V*. Using the same number of bins in each dimension for simplicity, H(V|U) is computed as

(11) $$H\left(V|U\right)=-\frac{1}{N}\sum_{i=1}^{Q}\sum_{j=1}^{Q}r_{i,j}\log\frac{r_{i,j}}{r_{j}},$$

where *i* and *j* indicate the indices of the bins along *V* and *U*, respectively, and $r_{i,j}$ is the number of observations that lie in the intersection of the bins *i* and *j*. TE can be computed by combining (11) and (5). More details about this method can be found in [13,15].

#### 2.3.4. Kernel Density Estimator

In the kernel density estimator (KDE), the PMF is estimated using a preselected distribution which is centered at each observation of the processes. All the distributions are summed to obtain an overall smooth distribution for the processes [13,19]. The shape of the distribution is defined by a kernel, which in this study corresponds to the widely used Gaussian kernel. The magnitude of this kernel decreases as the distance from the center increases. Hence, one of its main parameters is its bandwidth.

The probability mass for one observation $x_{t}$, using the KDE, is estimated as

(12) $$p\left(x_{t}\right)=\frac{1}{N}\sum_{i=1}^{N}\frac{1}{h}K\left(\frac{x_{t}-x_{t,i}}{h}\right),$$

where *K* is the kernel function with bandwidth *h*. The Gaussian kernel is defined as

(13) $$K\left(u\right)=\frac{1}{\sqrt{2\pi }}e^{-0.5u^{2}},$$

where $u=(\frac{x_{t}-x_{t,i}}{h})$, with the bandwidth of the kernel defined as

(14) $$h=1.06\alpha \hat{\sigma }N^{-\frac{1}{5}},$$

where α is a scaling multiplier and $\hat{\sigma }$ is the sample standard deviation.

Now, in the case of a given point ($x_{t},y_{t}$) in the joint space defined by the target and driver processes, the joint probability can be estimated as

(15) $$p\left(x_{t},y_{t}\right)=\frac{1}{N}\sum_{i=1}^{N}\frac{1}{h_{x_{t}}h_{y_{t}}}K\left(\frac{x_{t}-x_{t,i}}{h_{x_{t}}}\right)K\left(\frac{y_{t}-y_{t,i}}{h_{y_{t}}}\right).$$

Using (15) to compute H(X,Y) with (2), TE can be calculated with (4). More information about this method can be found in [13,19].

#### 2.3.5. Adaptive Partitioning Estimator

When working with binning methods for the estimation of PMFs, the fact of having a fixed number of bins with a defined and equal width can lead to an overestimation of the PMF. One solution to this issue is to use a non-uniform partition of the space defined by the observations of the processes [15,19]. This solution is implemented with the Darbellay–Vajda (DV) algorithm, combined with ordinal sampling [13].

For the adaptive partitioning (DVP) estimator, the ordinal sampling is performed as described for the FBR, replacing the time series of the observations of *X* and *Y* by *U* and *V*, respectively. The DV algorithm recursively partitions the three-dimensional space defined by *V*, $U^{-}$ and $V^{-}$ into cubes of different size, in order to obtain an even distribution of the data across the partitions. At first, the three-dimensional space is partitioned into eight equal cubes, of which the boundaries are at the mid-points in the three dimensions. The null hypothesis of an even distribution of the data points across the eight cubes is tested using a $\chi^{2}$ statistic,

(16) $$s_{\chi^{2}}=\sum_{i=1}^{8}{\left(M_{i}-\mu_{M}\right)}^{2},$$

where $M_{i}$ corresponds to the number of points contained in each of the eight cubes and $\mu_{M}$ is the total number of data points divided by the total number of cubes. If $s_{\chi^{2}}$ is greater than the $\chi^{2}$ statistic at a 5% significance level and 7 degrees of freedom, the null hypothesis is rejected, and each of the eight cubes is further partitioned into eight smaller cubes. If the null hypothesis is not rejected, the current cubes are taken as one partition.

The result of the recursion process is a finite number of cubes, *L*, with nonzero data points. The approximation of the probabilities for the computation of the entropies is done by counting the number of data points that are greater than or equal to the lower bounds and less than the upper bounds of each cube in each dimension, following (10). H(V|U) is estimated using (11) along all the cubes *L*. Finally, TE is computed using (5). More information on this method can be found in [13,15].

### 2.4. Simulation Study

Given the wide range of dynamics that are present in physiological systems, it is difficult to account for confounding effects. This makes the understanding and interpretation of their interactions more complex and the selection of a method for their study more difficult. Therefore, three simulation models were used to study the previously explained methods for the computation of TE. These simulation models vary in complexity, from a simple bivariate autoregresive (AR) linear model, to a bivariate model that includes linear and nonlinear components in its interactions. However, these models were not defined to represent a specific physiological phenomenon, but to illustrate different dynamics present in real data. For this reason, these results could be used in a broader context than physiological data.

For each simulation model, the performance of the methods was assessed using two analyses. For the first analysis (A1), the parameters of the methods to compute the TE were allowed to change. The effect of the selection of the lag (τ) for the generation of the embedding vectors was also assessed in this analysis. In the second analysis (A2), the strength of the interactions between the time series of each model was changed. An overview of the parameters of the methods that were changed in the analyses is presented in Table 1. The range in which the parameters change is based on the values used in previous studies [12,13,16,26,27].

#### 2.4.1. Simulation Models

For all simulation models described below, the variations of TE from the driver process *X* to the target process *Y* were analyzed. The TE from *Y* to *X* is expected to be equal to zero, since no influence of *Y* on *X* was imposed. A1 and A2 were performed on 50 trials of the models. Each trial corresponded to a new pair of signals *X* and *Y*, where the noise components are allowed to change. The length of the simulated signals was set to *N* = 200 samples. This length was selected so that it was comparable to the length of the clinical data, which ranges from 150 to 330 points.

##### Linear Model

The first simulation model corresponds to a linear AR bivariate Gaussian process of order 2 based on the work presented by Faes et al. [16], and defined as

(17) $$\begin{array}{cc} x_{n} & =-0.5x_{n-2}+\varepsilon_{x_{n}}\\ y_{n} & =-0.5y_{n-2}+ax_{n-1}+\varepsilon_{y_{n}}, \end{array}$$

where $\varepsilon_{x_{n}}$ and $\varepsilon_{y_{n}}$ are independent Gaussian white noise processes with zero mean and unit variance. The effect of *X* on *Y* is modulated by the parameter *a*.

For A1, the parameter *a* of the model is set to 0.5 and the time delay (τ) for the generation of the embedding vectors is changed between 1 and 5 (around the value at which the interaction takes place, τ=1). For A2, the parameter *a* is allowed to vary in the range from 0 to 0.5, while τ=1.

##### Nonlinear Model

The second simulation model is a bivariate process of order 2 that includes a nonlinear interaction. It is based on the work presented by Lee et al. [13], and defined as

(18) $$\begin{array}{cc} x_{n} & =s_{x_{n}}+\xi_{x_{n}}\\ y_{n} & ={\left(b\;x_{n-2}\right)}^{2}+\xi_{y_{n}}\\ s_{x_{n}} & ∼N(10,1)\\ \xi_{x_{n}} & ∼L\left(0,1\right),\;\xi_{y_{n}}∼L\left(0,1\right), \end{array}$$

where $\xi_{x_{n}}$ and $\xi_{y_{n}}$ correspond to noise components drawn from a Laplace distribution, and *b* is the coupling factor.

The parameter *b* is fixed at 0.4, while τ changes between 1 and 5 (around the value at which the interaction takes place, τ=2), for A1. For A2, the parameter *b* is allowed to change between 0 and 0.5, maintaining τ=2.

##### Linear + Nonlinear Model

The third simulation model is a bivariate system including a linear and a nonlinear term in *Y*. This model is adapted from the simulation study presented by Khadem and Hossein-Zadeh [22], and is defined as

(19) $$\begin{array}{cc} x_{n} & =0.3x_{n-1}+\varepsilon_{x_{n}}\\ y_{n} & =0.3y_{n-1}+cx_{n-2}+df\left(x_{n-4}\right)+\varepsilon_{y_{n}}\\ f(x_{n-4}) & =\frac{2.4-0.9(x_{n-4})}{1+\exp(-4\left(x_{n-4}\right))}, \end{array}$$

where $\varepsilon_{x_{n}}$ and $\varepsilon_{y_{n}}$ are independent Gaussian white noise processes with zero mean and unit variance, the function $f(x_{n-4})$ represents the nonlinear term, and the parameters *c* and *d* regulate the strength of the linear and nonlinear dynamics, respectively. $\tau_{l}=2$ and $\tau_{nl}=4$ correspond to the lags at which the linear and nonlinear interactions occur, respectively.

For A1, *c* and *d* were fixed at 0.4 and 0.6, respectively, while τ was changed from 1 to 5 (around the values at which the interactions take place, $\tau_{l}=2$ and $\tau_{nl}=4$). For A2, there were two set-ups for this model. The first one was for the linear interaction, having *c* varying between 0 and 0.6, while *d* was fixed at 0.4, and τ=2. The second one was for the nonlinear interaction, for which *c* was fixed at 0.6, and *d* changed from 0 to 0.6 and τ=4.

#### 2.4.2. Evaluation Quantities

For A1, the $TE_{X\overset{}{\to }Y}$ was expected to reach a maximum when using the lag at which the interaction between the signals occurs, and zero otherwise [32,33].

In order to evaluate the results of the methods in this case, a new index named Transfer Entropy Excess (TEE) is proposed. This index calculates the ratio between the total sum of the TE for all the lags and the TE at the lag at which the interaction occurs.

It is worth noting that this index can only be used with simulation studies for which the interaction lag is known, and it is not intended as a form to assess the validity of the TE computed on real data. TEE considers the possible over-estimation and intrinsic error of the methods when computing TE with different lags that would be also present when analyzing real data. It is defined as

(20) $$TEE=\frac{\sum_{i=1}^{T}TE_{X\overset{}{\to }Y}\left(\tau_{i}\right)}{TE_{X\overset{}{\to }Y}\left(\tau_{interaction}\right)},$$

where *T* is the total number of lags used in the analysis, $TE_{X\overset{}{\to }Y}\left(\tau_{i}\right)$ refers to the TE computed with the lag $\tau_{i}$, and $\tau_{interaction}$ is the τ of the interaction imposed in each simulation model. TEE can vary between 1 and infinite. The closer TEE is to 1, the better the method identifies the lag of the interaction. Higher values of TEE indicate that, for other lags, the TE is higher than zero, implying a possible bias of the method.

In A2, an increment in $TE_{X\overset{}{\to }Y}$ with stronger interactions between the signals was expected. To quantify the increment of TE as a function of the coupling parameter of the model, the linear regression of the TE on the coupling factor is used:

(21) $$TE_{X\overset{}{\to }Y}=\beta_{0}+\beta_{1}\omega ,$$

where $\beta_{0}$ is the intercept of the regression, $\beta_{1}$ is the slope of the regression, and ω is the coupling factor of each simulation model. The higher the slope, the higher the sensitivity of the method to identify changes in the interaction strength.

### 2.5. Application on Real Data

In this study, 1891 one-minute segments from PSG recordings of 26 subjects referred to the sleep laboratory of the University Hospitals Leuven, UZ Leuven, Belgium were used. The study was approved by the ethical committee of UZ Leuven (S53746, S60319) and all subjects signed an informed consent. The subjects considered for this study presented an apnea-hypopnea index (AHI) of less than 5. The median (25th; 75th) age and BMI of the subjects was 37 (34; 47) years, and 24.98 (23; 32.19) kg/m${}^{2}$. The data used in this study were collected for the OSA+ project, and have been published before in [34,35], where pulse photoplethysmography (PPG) and oxygen saturation (SpO${}_{2}$) were used in the detection of sleep apnea.

From the PSG, the ECG and respiratory signals were extracted (fs=500 Hz). Segments of one minute duration in which the subjects did not present apnea events from NREM1 and NREM3 were selected. The annotations of the respiratory events and hypnograms were done following the AASM2012 rules [36].

The ECG signal was filtered using a zero-phase Butterworth bandpass filter with cut-off frequencies at 0.03 and 150 Hz to remove baseline and high frequency noise. Then, it was filtered with a zero-phase Butterworth stopband filter at 50 Hz to remove powerline interferences. Next, the location of the R-peaks was found using R-DECO [37]. Missed, false and ectopic beats were corrected using the integral pulse frequency modulation (IPFM) model [38,39]. The corrected locations were then used to compute the time-series corresponding to time intervals between consecutive heart beats. This time series was resampled to 2 and 4 Hz to have an evenly sampled heart rate variability (HRV) representation. After that, a zero-phase Butterworth bandpass filter with cut-off frequencies at 0.03 Hz and 1 Hz was applied.

Two respiratory signals (RESP = nasal airflow (NAS) and respiratory effort measured around the thorax (THO)) were recorded using a pressure sensor and respiratory inductance plethysmography (RIP). These signals were first bandpass filtered using a zero-phase Butterworth filter with cut-off frequencies at 0.03 and 1 Hz. Then, they were resampled at 2 and 4 Hz. The reason to use two different sampling frequencies was to observe the effect that this has on the $TE_{RESP\overset{}{\to }HR}$ estimation. Previous works have found that the sampling frequency might influence the estimation of cardio-respiratory interactions [40], while other studies have found an effect of filtering and downsampling electrophysiological data on the estimation of TE between different brain areas [41].

#### 2.5.1. Significance Analysis

To validate the significance of the computed $TE_{RESP\overset{}{\to }HR}$, a surrogate data analysis was performed. Two approaches were used. The first one, known as iteratively refined surrogates (IRS), is applied to one signal at a time, and conserves the spectrum and distribution of the original data. This method guarantees that the generated surrogates of each signal maintain some of the characteristics of the original data, but that none of the interactions between them are preserved [42]. In the second approach, known as iterative multivariate surrogates (IMS), the surrogates are generated taking into account two or more signals at the same time. In this way, the distribution and spectrum of the original data and, additionally, the cross-correlation of the signals is preserved. The IMS will preserve the linear interactions of the original signals, but not the nonlinear interactions [42].

According to [42], to conduct a one-sided surrogate test at a significance level of 5%, at least 19 surrogate series are needed. Therefore, in the current work, for each segment, the $TE_{RESP\overset{}{\to }HRV}$ of the original data was compared against all of the $TE_{RESP\overset{}{\to }HRV}$ of 20 surrogates, generated with each approach. It is concluded that the $TE_{RESP\overset{}{\to }HRV}$ of the segment is significant if it is higher than the values obtained from all the surrogates.

By applying this methodology, it is possible to check whether linear or nonlinear interactions are present in the data. If the $TE_{RESP\overset{}{\to }HRV}$ of a segment is significant when using IMS, it can be concluded that the interaction between the signals includes a nonlinear component. On the other hand, if the $TE_{RESP\overset{}{\to }HRV}$ is significant when using IRS, the interaction between the signals could be either linear or nonlinear. In this case, if the $TE_{RESP\overset{}{\to }HRV}$ is also not significant using IMS, it can be concluded that the interaction is mostly linear. When the $TE_{RESP\overset{}{\to }HRV}$ is significant for IRS and IMS, it could be concluded that there is a nonlinear component in the interaction between the signals, but it is not possible to conclude anything about the linear component. These possible outcomes are shown in Table 2.

The explained framework for the analysis of TE on clinical data was applied to test the hypothesis that the linear and nonlinear interactions between RESP and HRV change in NREM1 and NREM3. TE estimates per sleep stage per subject were obtained by computing the median of the TE of the one-minute segments on which linear and nonlinear interactions were found to be significant.

## 3. Results

### 3.1. Simulation Study

For all the simulations, the five methods behaved similarly. However, FBR and KDE showed a positive bias consistently for all the simulations, due to the numerical approximations done when estimating the entropies. With KNN, some values of the TE were found to be negative, also because of the numerical approximations done by this method. LIN and DVP did not present any bias.

For A1, all the methods identified correctly the lag at which the interaction takes place, presenting a higher $TE_{X\overset{}{\to }Y}$ at this lag compared to the other lags. In the case of the linear model, additionally to the peak at τ=1, there were some smaller peaks at τ=3 and τ=5 for all methods. This was due to the autoregressive nature of the model, which included the influence of more past states of the driver process into the target process. In the case of the nonlinear model, there was only one peak in the TE, at τ=2, for all the methods. Finally, in the case of the linear + nonlinear model, there were two peaks present in the TE, given that, at τ=2 the linear interaction takes place and, at τ=4, the nonlinear interaction occurs.

**Figure 1:**
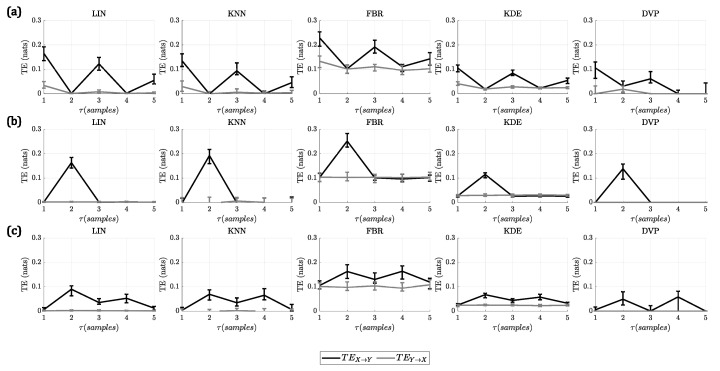
Results of changing the lag for the embedding vectors generation for (**a**) linear model (interaction at τ=1); (**b**) nonlinear model (interaction at τ=2); and (**c**) linear + nonlinear model (interaction at τ=2 and τ=4). Each plot shows the median TE vs. lag (τ) in samples. The error bars indicate the interquartile range. The columns show the methods, from left to right, LIN, KNN, FBR, KDE and DVP. It can be seen that all methods identify correctly the lag of the interaction. However, DVP presents the lowest over-estimation (bias) of TE for other lags in all the simulations.

In Figure 1, the results for this analysis are shown. For the first four methods (LIN, KNN, FBR, KDE), the results are shown for the best parameters. The complete results of changing all the parameters of these methods can be found in the Supplementary Material.

The TEE was calculated for all methods, and the parameters for which it was closer to 1 were selected and fixed to continue with A2. The best parameters for each method are presented in Table 3.

**Figure 2:**
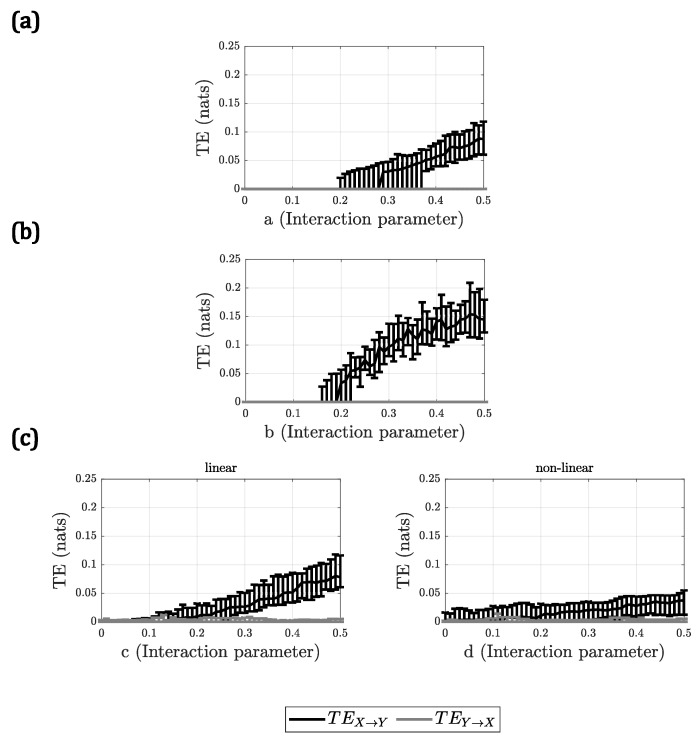
Results of DVP for changing the interaction strength for (**a**) linear model; (**b**) nonlinear model and (**c**) linear + nonlinear model. Each plot shows the median TE vs. the interaction parameter. The error bars indicate the interquartile range.

For A2, all methods behaved similarly, with the only difference being the slope of the TE with respect to the interaction parameter. In Figure 2, the results for A2 are presented, only for DVP as a representative for all the methods. Only in the case of the linear + nonlinear model, for the nonlinear interaction, the methods did not show a clear increment in TE when the interaction parameter *d* increased, demonstrating a very small slope in all cases.

A summary of the evaluation of the performance of the methods for all the simulation models is presented in Table 4. The best method corresponds to the one with the lowest TEE combined with the highest $\beta_{1}$.

Comparing the TEE values for all the methods, it could be seen that, for DVP, the TEE was lower and closer to 1 for all the models, indicating that it performed well when identifying the correct lag of the interactions. It could be noted that FBR had the highest values of TEE for all simulation models, which is consistent with the higher bias that this method presents, which is evident in Figure 1. The performance of LIN, KNN and KDE did not differ significantly from DVP; however, their TEE values are higher given the over-estimation of TE on other lags. After comparing the methods using the TEE index, it was found that DVP is the best method to identify the correct interaction lag, presenting the lowest bias.

When observing the $\beta_{1}$ values, it could be seen that all the methods behaved similarly. However, the methods that presented a slightly higher sensitivity to the change of the interaction strength were LIN and KNN. In this case, again FBR had the lowest performance, evidenced in the lowest values of $\beta_{1}$. KDE and DVP behaved comparably to LIN and KNN, only having slightly lower slopes in general. It could be noted that, for the nonlinear model, all the slopes were higher than for the other methods. In addition, in the case of the linear + nonlinear model, the slopes for the nonlinear interaction were the lowest, and the slopes for the linear interaction also remained lower than for the pure linear model. This could hint to the fact that, when there are multiple dynamics in a system, the sensitivity of the methods to identify each interaction is reduced.

In general, considering all the experiments, DVP can be identified as the best performing method. Hence, it was selected for the analysis in clinical data.

### 3.2. Application to Real Data

The analysis of the clinical data focused on the linear and nonlinear dynamics of the interactions between the respiration (RESP) and the heart rate variability (HRV) in two sleep stages, NREM1 and NREM3. Given that the delay at which the cardio-respiratory coupling occurs is not known, lags between 1 and 5 seconds were considered.

For each sleep stage, the significance of the TE was assessed using the surrogate analyses explained in Section 2.5.1. It is worth noting that, if the linear interaction is found to be significant in a segment, it means that the only component identified by TE was the linear one. Instead, if the nonlinear interaction is significant in a segment, it means that TE was able to identify a nonlinear component, but there could also be a linear component (see Table 2).

As mentioned in Section 2.5, a TE estimate for each patient and for each sleep stage was computed. This estimate corresponded to the median of all the segments with a significant interaction. In Table 5, the number of patients that presented significant interactions for each lag and sampling frequency are shown, when using each type of respiratory signal. The median TE values between sleep stages were compared using a Wilcoxon signed rank test with a significance level of 5%.

**Figure 3:**
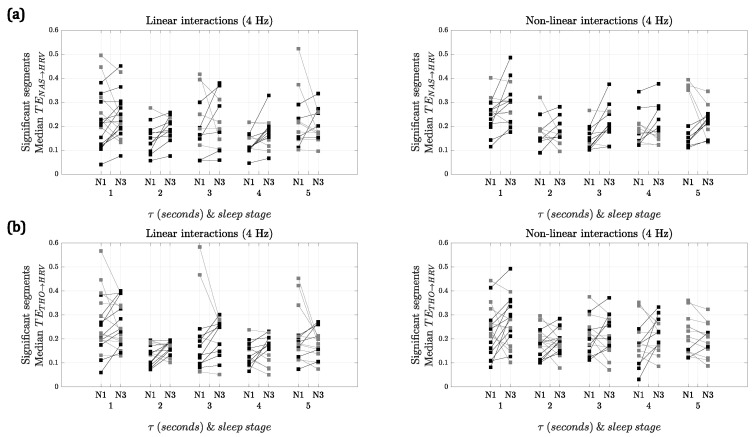
Median $TE_{RESP\overset{}{\to }HRV}$ of significant segments for each kind of interaction at 4 Hz vs. lag (τ) in seconds. (**a**) RESP=NAS; (**b**) RESP=THO. Gray lines represent higher interactions in NREM1, while black lines correspond to higher interactions in NREM3. Linear interactions are on the left, for which significant differences using NAS are found at τ=2 and τ=4 s. Nonlinear interactions are on the right, for which significant differences using NAS are found at τ=1 and τ=3 s. No significant differences are found using THO.

Figure 3 shows the median $TE_{NAS\overset{}{\to }HRV}$ and $TE_{THO\overset{}{\to }HRV}$ for the segments with significant interactions for both sleep stages using 4 Hz as the sampling frequency. Using NAS, when comparing the strength of the linear interactions, there was a significant difference between NREM1 and NREM3 at lags 2 and 4 s, with NREM3 being higher. In contrast, for the nonlinear interactions, the differences between both sleep stages were significant for lags 1 and 3 s. In the case of THO, no significant differences were found for any of the interactions.

For both of the respiratory signals, the results with 2 Hz (see Supplementary Material) were similar to the ones depicted in Figure 3. However, in the case of NAS, for the linear interactions, there was only a significant difference at lag 4 s, while, for the nonlinear interactions at lags 3, 4 and 5 s NREM3 presented higher TE. In the case of THO, for the linear interactions, only a significant difference at lag 2 s was found, while for nonlinear interactions there was a significant difference at lag 4 s.

It is worth noting that, even though NAS and THO represent the respiration of the subject, their morphologies are different and thus the results obtained are different. However, some similarities are found. For both sampling frequencies, more patients presented slightly higher values of TE in NREM3 for lags 2, 3 and 4 s in the linear interactions. For the nonlinear interactions, the trends are not consistent for both respiratory signals and sampling frequencies. The similarities and significance of these trends could be investigated further with more patients available for comparison.

## 4. Discussion

In this work, the application of TE for the analysis of different dynamics of interactions between time series was studied. For this purpose, five methods were applied to three simulation models with different types of interactions. It is worth noting that the simulation models used in this paper were not defined to represent the real physiological data of the cardiac and respiratory systems but were used to illustrate the different dynamics that could be present in real physiological data.

To generate the embedding vectors for the computation of TE, UE was used because of its simplicity. This technique, however, can be redundant and arbitrary in the selection of the embedding vectors, which could cause problems such as over-fitting and detection of false influences [31]. A possible approach to prevent these issues is to use a non-uniform embedding (NUE) technique. This approach was not covered in this study and its implementation with the entropy estimators is proposed as future work.

With TEE, it was possible to select the best parameters for each method, based on the knowledge of the exact lag at which the interaction between the signals occur. However, the best parameters found for this study are not meant to be used arbitrarily, as their selection can be affected by the length of the signals, as shown in [21] for mutual information. It is worth noting that TEE was meant to be used only in simulation studies, as for these the true interaction lag is known, which is a parameter for the computation of this index.

Out of the five methods compared in this work, only LIN was model-based, and made the assumption that the studied processes had a joint Gaussian distribution. This assumption, however, did not limit its performance when applied to nonlinear processes. The results obtained in this study, in which LIN performed similarly to the other methods, were contrary to the results of [12,26], in which LIN was outperformed by KNN. However, in [12,26], the signals analyzed were longer than the signals used in the current study. The limited number of points in the current study could have affected the accuracy of the estimation of the PMFs, which could explain the comparable performance of KNN and LIN. It is expected that, with a higher number of points, an increment in the accuracy of KNN when estimating the PMF could lead to a better performance of this method.

In Ref. [13], it was found that DVP outperformed FBR and KDE for nonlinear interactions, even though KDE was more sensitive to increments in the interaction strength. In this work, the same behavior was found, considering that DVP was the best method, not only compared with FBR and KDE, but also with LIN and KNN.

K-nearest neighbors and kernel based estimators were compared in [27], showing that the kernel estimators outperformed the k-nearest neighbor estimators. However, the kernel function used in [27] is different from the one used in this study, and the effect of the selection of the kernel function was not analyzed. In the current study, it was found that KNN presented a better behavior than KDE with a Gaussian kernel function.

In general, the finding that DVP outperformed all the other methods for the simulation models is consistent with the literature. Nevertheless, many studies use k-nearest neighbors or traditional binning estimators when applying TE and other information based metrics [14,18,43,44], due to their popularity and extensive use as non-parametric estimators. The current work could constitute a base for the selection of DVP as an alternative to the popular estimators, not only because of its performance, but also because having minimal adjustable parameters can constitute an advantage when working with real data. However, as future work, it is advised to extend the comparison of the methods presented in this study, and others that were not mentioned here, with simulation models that represent real physiological data and with longer signals. In addition, it is advised to explore the performance of the methods in non-stationary set-ups, relaxing the stationarity assumptions using a sliding window to obtain a continuous profile of the TE. This last approach could be useful when dealing with long and continuous measures of physiological data.

With the best method for the computation of TE identified, the cardio-respiratory interactions from segments of a PSG study were analyzed. It was decided to compute TE using lags between 1 and 5 seconds, since, during sleep, different forms of cardio-respiratory coupling could occur at different times [6], and the selection of the optimal embedding lag was out of the scope of this work.

For the study of these interactions, two types of respiratory signals were considered. The first one was the nasal airflow, and the second one was the respiratory effort measured around the thorax. The analysis included these two signals as their morphology is different, even though both represent the respiration of the patient and are recorded at the same time. It was found that the changes in the morphology of the respiratory signal affect the computation of TE, obtaining inconsistencies when comparing different lags and sampling frequencies.

In previous works, it has been suggested that the sampling frequency might influence the estimation of cardio-respiratory interactions [40]; therefore, two sampling frequencies were included in this analysis (2 Hz and 4 Hz). When observing the results for each type of respiratory signal, it was found that, even if the general behavior in terms of trends of the TE values between NREM1 and NREM3 was similar in each interaction, the significant differences were affected by the sampling frequency.

While the respiration and the HRV signals are highly regular during deep sleep for healthy subjects, the presence of sleep apneas could increase their variability. The higher variability could affect the resampling of the cardio-respiratory signals. In this case, it is possible that the information transferred from one signal to the other could not be quantified by TE. In Ref. [41], the effects of the downsampling and filtering of the driver and target signals on the computation of TE were studied, finding that the interactions were underestimated or not detected. These facts, combined with the difference in length between the signals, could explain the inconsistency between the results with both sampling frequencies in terms of significant differences.

It is also worth mentioning that, after selecting only the segments for which the linear and nonlinear interactions were found to be significant, not all patients were included in the final comparison. As showed in Table 5, the number of patients included in the significance analysis of the differences of TE between NREM1 and NREM3 varied from 7 to 20. The small size of these samples could have an effect in the significance test, and it is advised to explore further the hypothesis showed in the current study, including a larger pool of patients.

## 5. Conclusions

In this work, the behavior of five methods to compute TE was compared based on their performance on simulated data. With this comparison, the limitations and advantages of the methods when working with different dynamics and short signals were highlighted.

It was found that the DVP method performed best for the simulations. This method identified correctly the lag at which the interaction occurred for each simulation model, presenting lower values for different lags. The other methods presented higher TE values for different lags and, in the case of FBR and KDE, they had a constant bias for all lags. The performance regarding the sensitivity to the strength of the interactions showed that all methods were comparable. The lack of parameters to tune when using DVP was an advantage influencing its selection to be used with the cardio-respiratory signals from a PSG study.

For the clinical data, it was found that DVP was a suitable method to quantify the linear and nonlinear cardio-respiratory interactions during sleep. The results suggest that, for these signals, there is statistical evidence to hint that the linear and nonlinear interactions are higher for NREM3 at specific lags, depending on the respiratory signal used and the sampling frequency. Nevertheless, further research in this topic is proposed with bigger datasets.

The simulation study combined with the results from the clinical data could be helpful to select the best method to identify and quantify interactions between physiological signals, even when no knowledge about the nature of these interactions is available.

## Figures and Tables

**Table 1:** Parameters of methods for TE computation.

Method	Parameter	Range of Change	Source
LIN	regression model order	[1,2,3,4,5,6,7,8,9,10]	MuTE Toolbox [12]
KNN	number of neighbors (nn)	[5,10,15]	MuTE Toolbox
FBR	quantization levels (*Q*)	[4,6,8,10]	PhysioNet [13]
KDE	multiplier of kernel bandwidth (α)	[0.5,0.75,1,1.25,1.5]	PhysioNet
DVP	none		PhysioNet

**Table 2:** Possible outcomes of the significance tests with each surrogate generation methodology.

Result Significance Test with IRS ${}^{a}$	Result Significance Test with IMS ${}^{b}$	Linear Interaction	Nonlinear Interaction
NS ${}^{1}$	NS	No	No
S ${}^{2}$	NS	Yes	No
S	S	I ${}^{3}$	Yes

*^a^* IRS = Iteratively Refined Surrogates, *^b^* IMS = Iterative Multivariate Surrogates. ^1^ NS = Not significant, ^2^ S = Significant, ^3^ I = Inconclusive

**Table 3:** Best parameters of methods for TE computation.

Method	Parameter	Value
LIN	regression model order	linear model, nonlinear model, and linear+nonlinear model: 1
KNN	number of neighbors (nn)	linear model: nn=15, nonlinear model: nn=5, linear+nonlinear model: nn=15
FBR	quantization levels (*Q*)	linear model, nonlinear model, and linear+nonlinear model: Q=4
KDE	multiplier of kernel bandwidth (α)	linear model, nonlinear model, and linear+nonlinear model: α=1.5
DVP	none	

**Table 4:** Scores of TEE and $\beta_{1}$ for the assessment of the performance of the methods for each simulation model.

	Linear Model		Nonlinear Model		Linear + Nonlinear Model		
Method	TEE	$\beta_{1}$	TEE	$\beta_{1}$	TEE	$\beta_{1}\mathrm{Linear}$	$\beta_{1}\mathrm{Nonlinear}$
LIN	2.09	0.35	1.03	0.46	1.37	0.29	0.07
KNN	2.04	0.28	1.06	0.54	1.33	0.22	0.09
FBR	3.39	0.26	2.59	0.24	2.09	0.13	0.04
KDE	2.71	0.24	1.91	0.44	1.81	0.19	0.08
DVP	1.87	0.31	1	0.39	1.05	0.20	0.08

**Table 5:** Number of patients, out of 26, with significant linear and nonlinear interactions for each sampling frequency and lag.

	RESP=NAS				RESP=THO			
	2 Hz		4 Hz		2 Hz		4 Hz	
τ (seconds)	Linear	Nonlinear	Linear	Nonlinear	Linear	Nonlinear	Linear	Nonlinear
1	13	9	19	14	20	12	19	18
2	16	7	11	10	17	8	14	18
3	10	10	11	11	15	12	13	15
4	11	12	13	12	8	10	13	13
5	13	10	12	12	14	8	15	13

## Data Availability

The data presented in this study are available on request from the corresponding author.

## Supplementary Materials

The following are available online at https://github.com/AndreaRozo/Benchmarking-TE-Methods.git, Figures S1–S4: Results of changing the lag for the embedding vectors generation and the parameters of the LIN, KNN, FBR and KDE methods, Figure S5: Median $TE_{RESP\overset{}{\to }HRV}$ of significant segments for each kind of interaction at 2 Hz vs. lag (τ) in seconds, Tables S1–S4: Scores of TEE for the assessment of the best parameters for LIN, KNN, FRB and KDE for each simulation model.
